# Fermented *Portulaca oleracea* L. Juice: A Novel Functional Beverage with Potential Ameliorating Effects on the Intestinal Inflammation and Epithelial Injury

**DOI:** 10.3390/nu11020248

**Published:** 2019-01-23

**Authors:** Raffaella Di Cagno, Pasquale Filannino, Olimpia Vincentini, Vincenzo Cantatore, Ivana Cavoski, Marco Gobbetti

**Affiliations:** 1 Faculty of Sciences and Technology, Libera Università di Bolzano, 39100 Bolzano, Italy; raffaella.dicagno@unibz.it (R.D.C.); marco.gobbetti@unibz.it (M.G.); 2 Department of Soil, Plant and Food Science, University of Bari Aldo Moro, 70126 Bari, Italy; cantatore@outlook.it; 3 Unit of Human Nutrition and Health, Department of Food Safety, Nutrition and Veterinary Public Health, Istituto Superiore di Sanità, 00161 Roma, Italy; olimpia.vincentini@iss.it; 4 CIHEAM-MAIB, Mediterranean Agronomic Institute of Bari, 70010 Valenzano, Bari, Italy; cavoski@iamb.it

**Keywords:** *Portulaca oleracea*, lactic acid bacteria, functional beverage, antioxidant, intestinal inflammation, anti-inflammatory

## Abstract

*P. oleracea* L. contains high level of nutrients and biologically active compounds. Recently, lactic fermentation has been proposed as a biotechnological option to enrich the profile of biogenic compounds of *Portulaca oleracea* L. puree. This study investigated the capability of fermentation by selected lactic acid bacteria to enhance the restoring features of *Portulaca oleracea* juice towards intestinal inflammation and epithelial injury. Lactic acid fermentation markedly increased the total antioxidant capacity of *P. oleracea* juice, preserved the inherent levels of vitamins C, A, and E, and increased the bioavailability of the level of vitamin B_2_ and that of phenolics. The effects of fermented *P. oleracea* juice on a Caco-2 cell line were investigated using an in vitro model closest to the in vivo conditions. Fermented *P. oleracea* juice strongly decreased the levels of pro-inflammatory mediators and reactive oxygen species. It also counteracted the disruption of the Caco-2 cell monolayers treated with the inflammatory stimulus. We used a diversified spectrum of lactic acid bacteria species, and some effects appeared to be strains- or species-specific. Fermentation with *Lactobacillus kunkeei* B7 ensured the best combination for the content of bioactive compounds and the ability to counteract the intestinal inflammation and epithelial injury.

## 1. Introduction

Functional beverages represent the fastest-growing segment of the functional foods market [1]. In particular, the consumer demand for non-dairy beverages with high functionality is growing as a consequence of lifestyle choices (e.g., vegetarianism and veganism), the increase of adverse reactions to foods (e.g., food intolerance and malabsorption), and diet-related non-communicable diseases (e.g., cardiovascular disease) [2,3,4]. The development of fermented functional plant-based beverages is one of the most promising areas of research and innovation, addressed towards the set-up of fresh-like and ready-to-drink health-promoting beverages [1,4]. Lactic acid fermentation, as one of the most suitable tools to exploit the functional potential of plant matrices, improves the bioavailability and bioactivity of phytochemicals and enriches the plant matrices with functional bacterial metabolites [5]. Several vegetables and fruits, as well as medicinal plants, have been largely fermented by selected lactic acid bacteria [5]. *P. oleracea* L. has a huge potential as a raw substrate to develop novel functional foods, dietary supplements, or pharmaceutical preparations due to the high level of nutrients and biologically active compounds (e.g., polysaccharides, ω-3 and ω-6 fatty acids, cerebrosides, bioactive alkaloids, phenolics, carotenoids, and vitamins). Overlapping or complementary pharmacological effects (e.g., neuroprotective, antimicrobial, antidiabetic, antioxidant, anti-inflammatory, anti-ulcerogenic, and anticancer) define the biogenic profile of this plant substrate [6,7,8]. Ameliorating effects on inflammatory bowel diseases (IBS) symptoms through the modulation of the pro-inflammatory cytokines levels has been recently associated with the *P. oleracea* extracts [9]. Although their pharmacological effects are well documented [6,7,8], few studies have been addressed to exploit the use of *P. oleracea* as a functional beverage [10]. Recently, lactic fermentation has been proposed as a biotechnological option to enrich the profile of biogenic compounds of common purslane (*Portulaca oleracea* L.) [10]. Lactic fermentation was shown to enhance the levels of γ-aminobutyric acid (GABA) and linalool, in addition to modifying the profiles of alkaloids and polyunsaturated fatty acids [6]. Supported by these previous findings, our study exploited the same fermentation protocol to improve the restoring effects of *P. oleracea* juice towards intestinal inflammation and epithelial injury. Microbial metabolites may down-regulate the pro-inflammatory response by the intestinal epithelial cells attenuating the symptoms associated with the IBS [11,12]. The effects of fermented *P. oleracea* juice on a Caco-2 cell line have been investigated using an in vitro model closest to the in vivo conditions. After confluence, Caco-2 cells differentiate structurally and functionally into enterocyte-like cells representing a suitable model to assess the physiological response of intestinal mucosa to oxidative stress and inflammatory status [11,13,14,15,16]. The protective action of fermented *P. oleracea* juice has been evaluated as the ability to modify the cellular redox status (production of reactive oxygen species), to modulate the secretion of pro-inflammatory mediators (prostaglandin E2, nitric oxide, proinflammatory cytokines), and to counteract the disruption of epithelial integrity.

## 2. Materials and Methods 

### 2.1. Microorganisms and Culture Conditions

Eight strains of lactic acid bacteria, see Table 1, belonging to the Culture Collection of the Department of Soil, Plant, and Food Science, University of Bari Aldo Moro, Bari, Italy, were used as starters for fermentation. Strains were identified by partial sequencing of the 16S *rRNA*, *recA*, *pheS*, and *rpoA* genes. Cultures were maintained as stocks in 15% (*v*/*v*) glycerol at −80 °C and routinely propagated at 30 °C for 24 h in de Man Rogosa Sharp (MRS) broth (Oxoid, Basingstoke, Hampshire, UK). Sequences have been deposited in GenBank under the accession numbers given in Table 1. Strains were previously isolated from fruits, vegetables, or honeybee-gut and characterized for technology (e.g., acidifying capacity) and functional features [17,18,19,20,21,22,23]. Their aptitude to ferment a *Portulaca oleracea* L. puree was also preliminarily verified by Filannino et al. [10].

### 2.2. Portulaca Oleracea L. juice (PJ) Processing 

The aerial parts of the *P. oleracea* L. plant were collected in September 2017 from the North of the Apulia region (Italy). A botanist confirmed the identification. After harvesting, the aerial parts were washed with water, cut into small pieces, and blended with a Classic Blender 400 (PBI International, Milan, Italy). Juice was collected from *P. oleracea* puree by centrifugation (10,000× *g*, 20 min, 4 °C) and sterilized by filtration on 0.22 μm membrane filters (Millipore Corporation, Bedford, MA, USA). The sterility of the resulting *P. oleracea* juice (PJ) juice was confirmed through plate counts. PJ was fermented under a standardized protocol previously described by Filannino et al. [10]. Lactic acid bacteria strains were used singly as starters. Cells were cultivated in MRS broth until the late exponential growth phase was reached (ca. 12 h), washed twice in 50 mM phosphate buffer, pH 7.0, and re-suspended into PJ at the initial cell density of ca. Log 7 CFU mL^−1^. PJ was incubated at 30 °C for 36 h. Growth was monitored by plating on MRS agar (Oxoid, Basingstoke, Hampshire, England) at 30 °C. The pH was measured by a Foodtrode electrode (Hamilton, Bonaduz, Switzerland). Samples were taken before (raw PJ) and after fermentation. PJ, without bacterial inoculum and chemically acidified with lactic acid (final pH of ca. 4.0), was incubated under the same conditions and used as control (CA–PJ). PJs were stored at −20 °C until analyses. 

### 2.3. Antioxidant In Vitro Assays

Free radical scavenging capacity was determined through DPPH**˙** and ABTS**˙** radical scavenging activity assays and ferric reducing antioxidant power (FRAP) assay. All analyses were carried out on fermented PJ, Raw-PJ, and CA-PJ. DPPH**˙** radical scavenging activity was measured by using the stable 1,1-diphenyl-2- picrylhydrazyl radical (DPPH**˙**) as reported by Brand-Williams et al. [24]. A quantity of 10 µL of PJ were added to 3 mL of 40 μM DPPH solution and mixed. Samples were kept in dark conditions for 60 min at room temperature and the reaction was monitored by reading the absorbance at 517 nm. Results were shown as Trolox mmol equivalents per 100 mL of PJ. ABTS assay is based on the oxidation of the 2,20-azinobis-(3-ethylbenzothiazoline-6-sulphonic acid) di-ammonium salt (ABTS) by potassium persulphate to form a radical cation (ABTS**˙**) [25]. The reaction mixture was prepared by mixing 7.0 mM ABTS solution and 4.95 mM potassium persulfate solution in a ratio of 1:2. The mixture was allowed to react for 12 h at room temperature under dark conditions. A quantity of 1 mL of the mixture was diluted with 60 mL of methanol to obtain an absorbance of 0.70 ± 0.02 at 734 nm. PJ (20 µL) was allowed to react with 2 mL of the reaction mixture for 1 min under dark conditions. The reaction was monitored by reading the absorbance at 734 nm. Results were shown as Trolox mmol equivalents per 100 mL of PJ. A FRAP assay was performed according to Benzie and Strain [26] with some modifications. The reaction mixture was prepared by mixing 200 mL of 300 mM acetate buffer (3.1 g sodium acetate and 16 mL glacial acetic acid, pH 3.6), 20 mL of 10 mM 2,4,6- tripyridyl-s-triazine solution in 40 mM HCl, and 20 mL of 20 mM ferric chloride solution. The mixture was heated in a water bath at 50 °C, and PJ (20 µL) was allowed to react with 2 mL of the reaction mixture for 4 min under dark conditions. The reaction was monitored by reading the absorbance at 593 nm. Results were shown as Fe(II) mol equivalents per 100 mL of PJ. 

### 2.4. Vitamins Determination

HPLC determination of vitamin C, B_1,_ B_2_, B_3_, A, and E followed the EN 14130:2003, EN 14122:2003, EN 14152:2003, EN 15652:2009, EN 12823-2:2000, and EN 12822:2000 standard methods, respectively [27,28,29,30,31,32]. The quantity of vitamins was expressed as mg per 100 mL of PJ and was determined as mg of ascorbic acid, thiamin, riboflavin, niacin, β-carotene, and α-tocopherol for vitamin C, B_1,_ B_2_, B_3_, A, and E, respectively. 

### 2.5. Total Phenolics Determination

Total phenolic compounds were assayed according to the Folin–Ciocalteu method [33,34]. Data were expressed as gallic acid mg equivalents per 100 mL of PJ.

### 2.6. Caco-2 Cell Culture

Human colon carcinoma Caco-2 cells (ATCC, passage 32) were routinely cultured in 25 cm^2^ culture flasks (BD Biosciences, New Jersey, USA) in Dulbecco’s modified Eagle’s medium (DMEM) high glucose supplemented with 10% (*v*/*v*) heat-inactivated FBS, 1% (*v*/*v*) HEPES 1M, 1% (*v*/*v*) non-essential amino acids, 1% L-glutamine 200 mM, penicillin (100 U mL^−1^), and streptomycin (100 μg mL^−1^) (all from Life Technologies, Grand Island, NY, USA) [13]. Cells were kept in an incubator at 37 °C in a 5% CO_2_ and 95% air-humidified atmosphere. The medium was refreshed every 2–3 days and cells were split once a week. The cytotoxicity of PJ was examined by the use of the MTT assay, incubating Caco-2 cells with freeze-dried PJ powder (1–500 μg mL^−1^) for 24 h. Cytotoxicity was observed with a concentration ≥ 500 μg mL^−1^. Thus, 100 μg mL^−1^ of freeze-dried PJ powder was used as the maximum dose.

### 2.7. Secretion of Pro-Inflammatory Mediators

For cell treatments, freeze-dried PJ powder was resuspended in DMEM (10 mg mL^−1^, stock solution) and sterilized through 0.22 mm filter membrane (Millipore Corporation, Bedford, MA, USA) to remove lactic acid bacteria cells. Caco-2 cells were seeded onto 24 wells at a density of 10 × 10 ^3^ cells and cultivated for 21 days to allow differentiation. Cells were pretreated for 6 h with freeze-dried PJ powder (100 μg mL^−1^) before the inflammatory stimulus was added and then incubated for others 24 h. 

The inflammatory stimulus for prostaglandin E2 (PGE2) and nitric oxide was represented by a cytokine mix (LPS, 10 ng mL^−1^; TNF-α, 50 ng mL^−1^; IL-1β, 25 ng mL^−1^). Interleukin-1β (IL-1β) was used at the concentration of 25 ng mL^−1^ for monocyte chemotactic protein-1 (MCP-1) and interleukin-8 (IL-8) assay. When added alone, cytomix was used as a positive control. After treatment, the culture supernatant was collected, and PGE2, MCP-1, and IL-8 were analyzed after centrifugation at 1500 rpm for 5 min and determined using a commercial enzyme-linked immunosorbent assay (ELISA) kits according to the manufacturer’s instructions [35,36]. Each kit was confirmed for their sensitivity, recovery, linearity, and precision. For precision, inter- and intra-assays were performed by the manufacturer. A PGE2 Elisa Kit was from Mybiosource (San Diego, CA, USA.) and IL-8 and MCP-1 were from Biomatik (Ontario, Canada). The concentrations of PGE2, MCP-1, and IL-8 in the culture supernatant were read by a spectrophotometer at 450 nm (Biorad, Hercules, USA). For nitric oxide (NO) release evaluation, cells treatment was carried out in DMEM without phenol red. At the end of the incubation, the cell culture supernatant was mixed with Griess reagent (1% sulfanilamide, 0.1% naphthyletylendiamine dihydrochloride, and 2% phosphoric acid) and the absorbance was measured at 540 nm using a microplate reader (Biorad, Hercules, CA, USA) [37]. 

### 2.8. Intracellular Reactive Oxygen Species (ROS)

The intracellular generation of ROS was assessed by measuring the oxidation of the probe 20,70-dichlorofluorescin diacetate (DCFH-DA) (Molecular Probes, Lifesciences) [38]. Briefly, the confluent Caco-2 cells in the 24-well plates were pretreated for 18 h with freeze-dried PJ powder (100 μg/mL). A set of the samples were treated with 10 μM H_2_O_2_ 2 h before the end of the treatment to induce oxidative stress, and the fluorescence intensity of DCFH-DA (relative fluorescence units) was measured at an excitation and emission wavelength of 485 nm and 520 nm, respectively, using a spectrofluorimeter (Victor3, Perkin-Elmer, Waltham, MA, USA). 

### 2.9. Measurement of Intestinal Barrier Function

Caco-2 cells were seeded at a density of 2.5 × 10^6^ cells on polycarbonate inserts (BD Biosciences, San Jose, CA, USA) and kept in the culture for up to 21 days to allow differentiation. The intestinal barrier function was evaluated by measuring the transepithelial electrical resistance (TEER) using a Millicell ERS device (Millipore, Bedford, MA, USA) [39]. Cells were left to differentiate and resistance values were registered. Only cells with approximately 800 Ω × cm^2^ were used. Cells were exposed to PJ for 6 h before adding the inflammatory stimulus IL-1β (25 ng mL^−1^) and monitored for 24 h. DMEM containing IL-1β alone was used as a positive control. Cells were allowed to settle for 10 min at room temperature before each measurement to enable the electrodes to equilibrate with external temperature. The effect of each sample on the cell monolayer was expressed as the TEER value relative with respect to the value at time zero. The background resistance was determined by measuring the resistance from cell-free inserts. 

### 2.10. Statistical Analysis

Analyses were carried out in triplicate with three biological replicates for each condition. Data were subjected to an analysis of variance (ANOVA) test for multiple comparisons (one-way ANOVA followed by Tukey’s procedure at *p* < 0.05), using the statistical software, Statistica 7.0 (StatSoft, Palo Alto, CA, USA). 

Normalized data for antioxidant activities, levels of bioactive compounds, PGE2, MCP-1, IL-8, NO, ROS, and TEER were subjected to permutation analysis using PermutMatrix (version 1.9.3).

## 3. Results

### 3.1. Portulaca Oleracea L. Juice (PJ) Processing

Eight lactic acid bacteria strains were singly used as starters to ferment PJ for 36 h at 30 °C. The aptitude of the strains, as well as the process parameters, were previously set up by Filannino et al. [10]. The initial cell density of all inoculated batches was ca. 7.0 log cfu mL^−1^. After 36 h of fermentation, the highest cell densities were found in PJ fermented with *Lactobacillus plantarum* strains (8.30–8.42 log cfu mL^−1^), whereas that of the other strains was slightly lower and ranged from 8.07 ± 0.12 to 8.25 ± 0.14 log cfu mL^−1^. Raw PJ had an initial pH value of 5.08 ± 0.03. After fermentation, the lowest (*p* < 0.05) value of pH were found with *L. plantarum* strains (4.10–4.15 pH units), followed by *Pediococcus pentosaceus* CILSWE5 (4.23 ± 0.02 pH units), and *Lactobacillus rossiae* 2MR8 (4.26 ± 0.03 pH units). For the other strains, only a slight variation of ca. 0.3 units was found. 

### 3.2. In Vitro Antioxidant Activity

**Figure 1:**
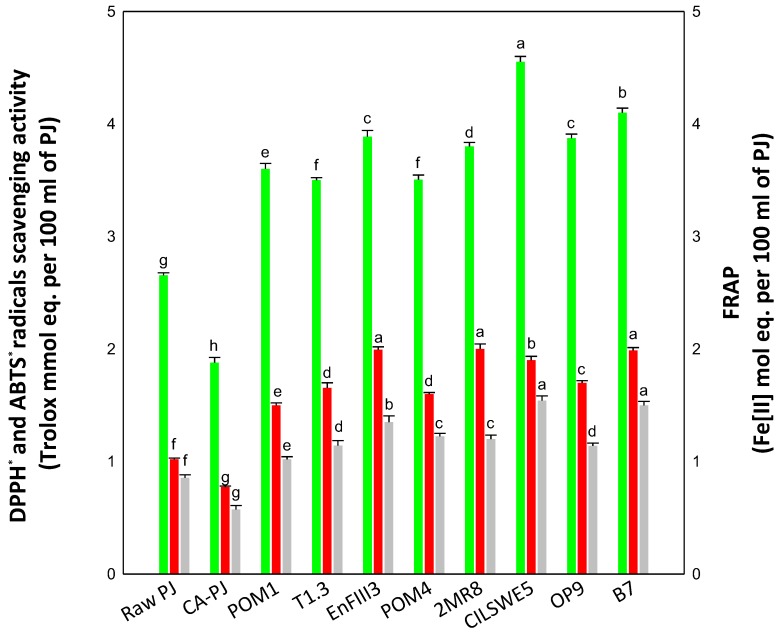
DPPH**˙** (green bars) and ABTS**˙** (red bars) radicals scavenging activities, and ferric reducing antioxidant power (FRAP) (grey bars) of raw *Portulaca oleracea* L. juice (Raw-PJ), chemically acidified PJ (CA-PJ), and PJs fermented at 30°C for 36 h by *Lactobacillus plantarum* POM1, T1.3, and EnFIII3, *Lactobacillus brevis* POM4, *Lactobacillus rossiae* 2MR8, *Pediococcus pentosaceus* CILSWE5, *Leuconostoc mesenteroides* OP9, and *Lactobacillus kunkeei* B7. Bars with the same colors and different superscript letters differ significantly (*p* < 0.05).

Due to the many involved variables, the measurement of the antioxidant capacity of food cannot be evaluated satisfactorily using a single antioxidant assay. In this study, we determined the antioxidant activity of PJ by using DPPH**˙**, ABTS**˙**, and FRAP assays. Raw-PJ showed a high inherent antioxidant activity, as estimated through the DPPH**˙** (2.66 ± 0.02 Trolox mmol eq. per 100 mL of PJ), ABTS**˙** (1.02 ± 0.01 Trolox mmol eq. per 100 mL of PJ), and FRAP assays (0.86 ± 0.03 Fe(II) mol eq. per 100 mL of PJ). Regardless of the assay and the bacterial starter, fermentation markedly increased (*p* < 0.05) the antioxidant activity of PJ, see Figure 1. Compared to Raw-PJ, the antioxidant activity of fermented PJs increased from 32 to 96%, as shown in Figure 1, where the highest increases (*p* < 0.05) were mainly observed when PJ was fermented with *P. pentosaceus* CILSWE5 (71%, 86%, and 79% as estimated through the DPPH**˙,** ABTS**˙**, and FRAP assays, respectively), *L. kunkeei* B7 (54%, 95%, and 74%), *L. plantarum* EnFIII3 (46%, 96%, and 57%), and *L. rossiae* 2MR8 (43%, 96%, and 40%), see Figure 1. Conversely, a decrease in the antioxidant activity was found in CA-PJ, see Figure 1. 

### 3.3. Vitamins and Phenolics Determination

Ascorbic acid (vitamin C) was found as the most abundant vitamin in Raw–PJ (22 ± 2 mg per 100 mL of PJ), followed by α-tocopherol (vitamin E) (2.5 ± 0.1 mg per 100 mL of PJ), and β-carotene (vitamin A) (2.00 ± 0.02 mg per 100 mL of PJ), see Table 2. Niacin (vitamin B_3_), riboflavin (vitamin B_2_), and thiamin (vitamin B_1_) were found at concentrations lower than 1 mg per 100 mL of PJ. Compared to Raw-PJ, reduced amounts of ascorbic acid were detected in CA-PJ and fermented PJs, except for those fermented with *L. kunkeei* B7, *L. brevis* POM4, and *L. plantarum* POM1 where the concentration remained unchanged, see Table 2. The same preserving effect was found for β-carotene when PJs was fermented with *L. kunkeei* B7 and *L. brevis* POM4. Apart from PJs fermented with *L. plantarum* POM1 and EnFIII3, a decreasing trend (*p* < 0.05) for the niacin was found in almost all fermented PJs and CA-PJ, see Table 2. The level of α-tocopherol decreased (*p* < 0.05) only in CA-PJ, whereas no significant change (*p* > 0.05) was found for thiamin, see Table 2. Compared to the inherent level of the Raw-PJ, the fermentation increased (*p* < 0.05) the riboflavin content, especially with *L. plantarum* POM1 and EnFIII3, and *L. kunkeei* B7 (67–56%), whereas no change (*p* > 0.05) was found in CA-PJ, see Table 2.

The amount of total phenolics in Raw-PJ was 85 ± 6 gallic acid mg equivalents per 100 mL of PJ. Overall, an increasing trend was observed for the total phenolics in fermented PJs, especially with* P. pentosaceus* CILSWE5 (ca. 71%), whereas a significant (*p* < 0.05) decrease (ca. 18%) was found in CA-PJ, see Table 2.

### 3.4. Viability of Caco-2 Cells

The cytotoxicity of PJs was checked using the MTT assay. Apart from the lactic acid bacterium used for fermentation, PJs behaved similarly to DMEM (negative controls). The concentration of freeze-dried PJ powder < 500 μg mL^−1^ did not significantly (*p* > 0.05) affect the Caco-2 cell proliferation.

### 3.5. Secretion of Pro-Inflammatory Mediators

**Figure 2:**
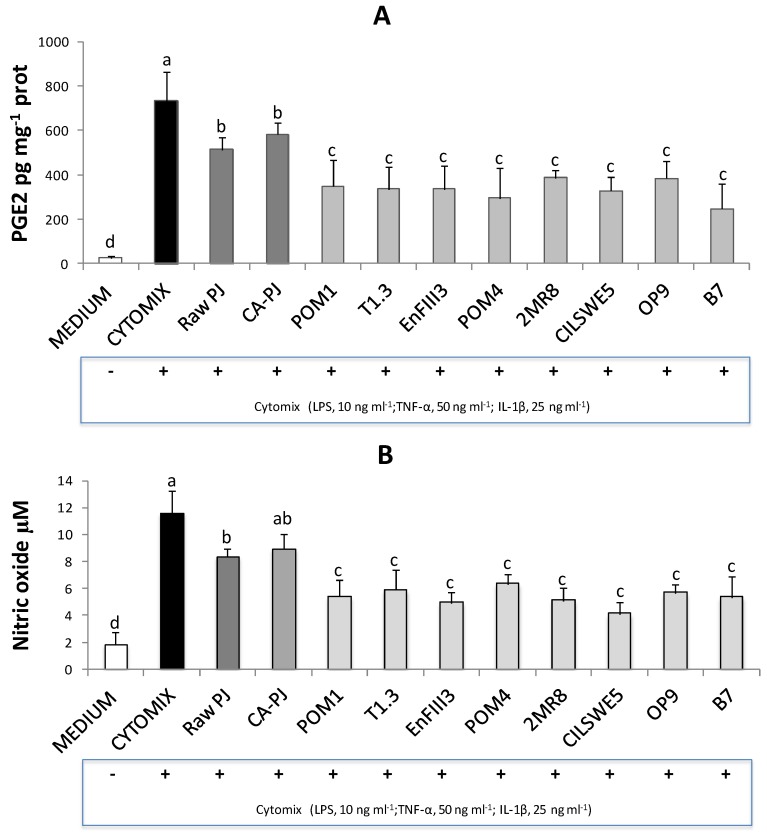
Prostaglandin E2 (PGE2) (pg mg^−1^ of protein) (A) and nitric oxide (NO) (µM) (B) levels measured in Caco-2 cells pretreated for 6 h with raw *Portulaca oleracea* L. juice (Raw-PJ), chemically acidified PJ (CA-PJ), and PJs fermented (30 °C for 36 h) by *Lactobacillus plantarum* POM1, T1.3, and EnFIII3, *Lactobacillus brevis* POM4, *Lactobacillus rossiae* 2MR8, *Pediococcus pentosaceus* CILSWE5, *Leuconostoc mesenteroides* OP9, and *Lactobacillus kunkeei* B7, then stimulated with the inflammatory cytomix for 24 h. Bars with different superscript letters differ significantly (*p* < 0.05).

Pro-inflammatory mediators play important roles in regulating the inflammatory response. The formation of prostaglandin E2 (PGE2), which is synthesized from arachidonic acid by cyclooxygenase enzymes, was determined. Treatment of Caco-2 cells with the cytomix solution including LPS, TNF-α, and IL-1β markedly stimulated (*p* < 0.05) the synthesis of PGE2 (734 ± 134 pg mg^−1^ of protein) and the release of NO (11.6 ± 1.7 µM) compared to the negative control (DMEM) (26 ± 7 pg mg^−1^ of protein and 1.8 ± 1.0 µM, respectively), see Figure 2A. The incubation with PJs significantly (*p* < 0.05) counteracted PGE2 accumulation in response to the cytomix. Raw-PJ and CA-PJ led to a decrease of PGE2 levels of ca. 30% and 20%, respectively. The highest (*p* < 0.05) decrease (47–66%) was observed with fermented PJs, see Figure 2A. A similar trend was found for the NO release as a response to the cytomix stimulation. Incubation with fermented PJs led to a decrease (*p* < 0.05) of NO levels of 45–65%, whereas only a weak decrease (*p* < 0.05) was observed with Raw-PJ and CA-PJ (28% and 23%, respectively), see Figure 2B. 

**Figure 3:**
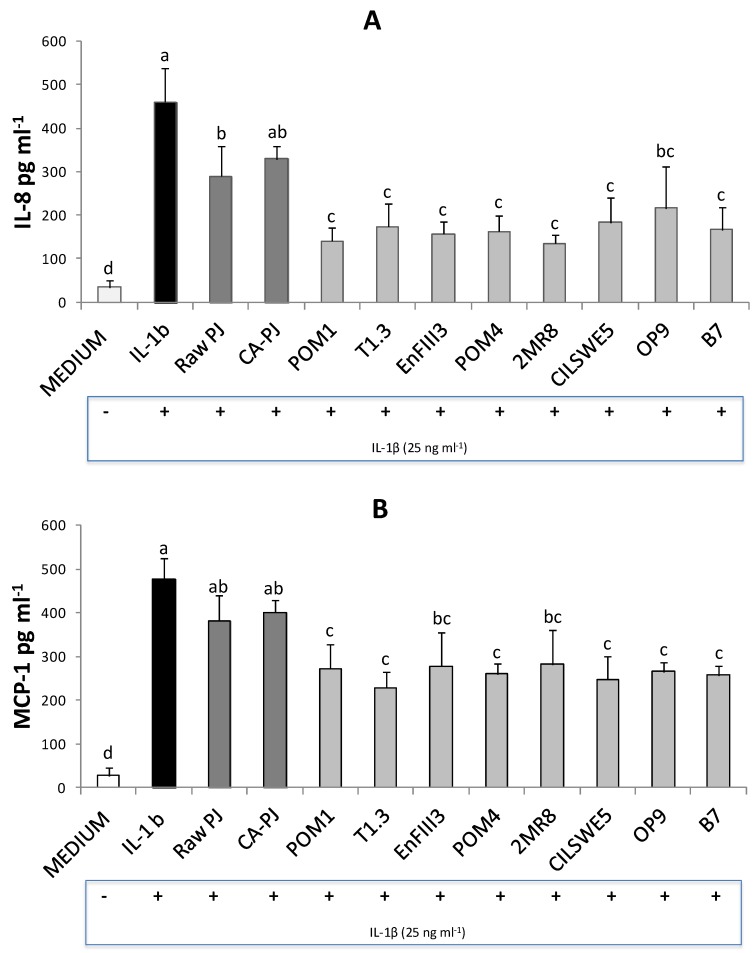
Interleukin-8 (IL-8) (A) and monocyte chemotactic protein-1 (MCP-1) (B) levels (pg mL^−1^) measured in Caco-2 cells pretreated for 6 h with raw *Portulaca oleracea* L. juice (Raw-PJ), chemically acidified PJ (CA-PJ), and PJs fermented (30 °C for 36 h) by *Lactobacillus plantarum* POM1, T1.3, and EnFIII3, *Lactobacillus brevis* POM4, *Lactobacillus rossiae* 2MR8, *Pediococcus pentosaceus* CILSWE5, *Leuconostoc mesenteroides* OP9, and *Lactobacillus kunkeei* B7, then stimulated and treated with the inflammatory stimulus (Interleukin-1β 25 ng mL^−1^) for 24 h. Bars with different superscript letters differ significantly (*p* < 0.05).

ELISA analysis revealed significant increases in levels of cytokines interleukin-8 (IL-8) and monocyte chemotactic protein-1 (MCP-1) (pg mL^−1^) in Caco-2 cells stimulated with Interleukin-1β (IL-1β) (461 ± 78 and 478 ± 49 pg mL^−1^, respectively) compared to the negative control (DMEM) (34 ± 16 and 27 ± 18 pg mL^−1^, respectively), see Figure 3. The levels of IL-8 significantly (*p* < 0.05) decreased when Caco-2 cells were treated with the fermented PJs (53–71%), and to a lesser extent with Raw-PJ (37%), see Figure 3A. No significant change (*p* > 0.05) was found with CA-PJ, see Figure 3A. Compared to the levels of MCP-1 measured in cells treated with IL-1β, only the treatment with fermented PJs led to a significant (*p* < 0.05) decrease (41–52%), see Figure 3B. No significant changes (*p* > 0.05) were found with Raw-PJ and CA-PJ, see Figure 3B.

### 3.6. Intracellular Reactive Oxygen Species (ROS)

**Figure 4:**
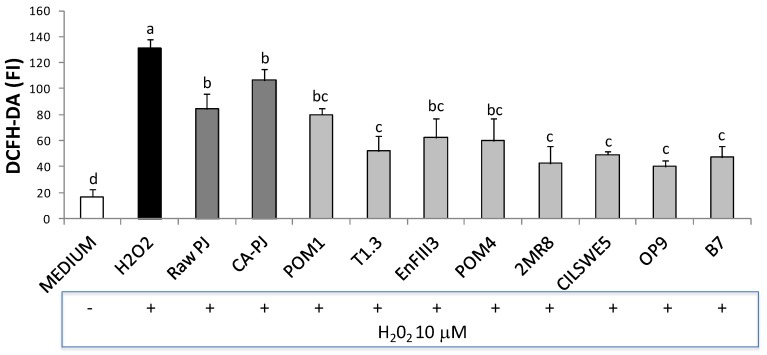
Intracellular reactive oxygen species (ROS) level in Caco-2 cells, measured as the fluorescence intensity (FI) of 20,70-dichlorofluorescin diacetate (DCFH-DA). Caco-2 cells were pretreated for 18 h with raw *Portulaca oleracea* L. juice (Raw-PJ), chemically acidified PJ (CA-PJ), and PJs fermented (30 °C for 36 h) by *Lactobacillus plantarum* POM1, T1.3, and EnFIII3, *Lactobacillus brevis* POM4, *Lactobacillus rossiae* 2MR8, *Pediococcus pentosaceus* CILSWE5, *Leuconostoc mesenteroides* OP9, and *Lactobacillus kunkeei* B7. Two hours before the end of the treatment, Caco-2 cells were treated with 10 µM H_2_O_2_ to induce oxidative stress. Bars with different superscript letters differ significantly (*p* < 0.05).

Intracellular ROS accumulation in Caco-2 cells is shown in Figure 4. ROS generation significantly (*p* < 0.05) increased when the cells were treated with H_2_O_2_ (631 ± 6 fluorescence units) compared to the negative control (DMEM) (17 ± 6 fluorescence units). When the cells were pretreated with fermented PJs and subsequently exposed to oxidative stress (+H_2_O_2_), ROS level was significantly (*p* < 0.05) decreased up to 70%, whereas a decrease of 36% and 19% was observed when the cells were pretreated with Raw-PJ and CA-PJ, respectively, see Figure 4.

### 3.7. Measurement of Intestinal Barrier Function by Determination of Transepithelial Electrical Resistance (TEER)

**Figure 5:**
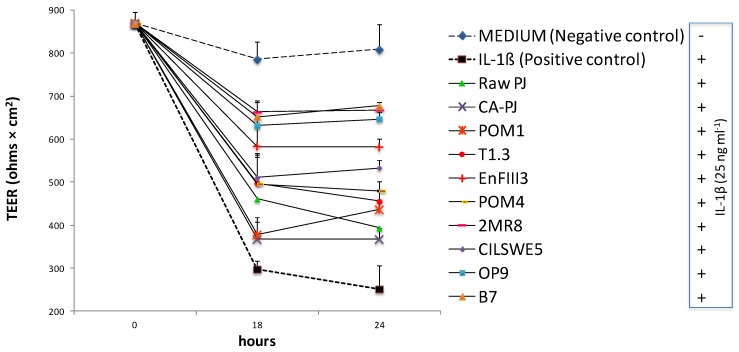
Transepithelial electric resistance (TEER) (Ohms × cm^2^) of Caco-2 cells. Caco-2 cells were pretreated for 6 h with raw *Portulaca oleracea* L. juice (Raw-PJ), chemically acidified PJ (CA-PJ), and PJs fermented (30 °C for 36 h) by *Lactobacillus plantarum* POM1, T1.3, and EnFIII3, *Lactobacillus brevis* POM4, *Lactobacillus rossiae* 2MR8, *Pediococcus pentosaceus* CILSWE5, *Leuconostoc mesenteroides* OP9, and *Lactobacillus kunkeei* B7, and incubated 24 h with interleukin 1β (IL-1β) (25 ng/mL). Data are the means (± SD) of three biological replicates analyzed in triplicate.

TEER is a widely accepted method to quantitatively measure the dynamics of epithelial integrity through an in vitro model. Compared to the negative control (DMEM), IL-1β induced a significant (*p* < 0.05) decrease of TEER (ca. 69%) in Caco-2 cells during 24 h of incubation, see Figure 5. However, pretreatment of cells with PJs exerted a protective effect against the induced decrease of TEER, see Figure 5. After 24 h of incubation with IL-1β, the lowest (*p* < 0.05) decrease of TEER was found when PJs was fermented with L. kunkeei B7 (ca. 16%), L. rossiae 2MR8 (ca. 17%), and Leuc. mesenteroides OP9 (ca. 20%), see Figure 5. The protective effect was less effective when Caco-2 cells were treated with Raw-PJ and CA-PJ leading to a decrease of TEER of ca. 51 and 55%, respectively, as shown in Figure 5.

## 4. Discussion

In the last few years, many plant active compounds with antioxidant and anti-inflammatory activities have been shown to exert potential clinical applications for the treatment of the chronic inflammatory bowel syndrome (IBS) [9,40,41,42,43,44,45,46]. IBS refers to several chronic inflammatory disorders of the gastrointestinal tract triggered by the action of environmental factors in genetically predisposed individuals [47]. The prevalence of IBS is highest in Europe followed by North America, although its incidence is also increasing in populations previously considered to be low risk, such as in Japan and India [48,49,50]. Although there is a low number of relevant clinical studies, the successful application of phytochemicals for IBS treatment seems to be promising [48]. Since the potential effectiveness of *Portulaca oleracea* extracts has been reported [9], this study investigated the lactic acid fermentation as a natural strategy to improve the restoring effects of *P. oleracea* L. juice towards intestinal inflammation and epithelial injury. Eight strains of lactic acid bacteria, previously successfully used to carry out the fermentation of *P. oleracea* puree, were individually used as starters to ferment the *P. oleracea* juice [10]. 

First, lactic fermentation markedly increased the total antioxidant capacity of *P. oleracea* juice, mainly when *Lactobacillus kunkeei* B7 and *Pediococcus pentosaceus* CILSWE5 were used as starters. Reactive oxygen species (ROS) have been suggested as key molecules in mediating the tissue injuries promoted by the inflammatory processes occurring in IBS [51,52,53,54]. Human’s endogenous antioxidant defense systems are incomplete without exogenous originating reducing compounds, such as vitamins and phenolics, and the gastrointestinal tract represents the major site of interaction between endogenously generated ROS and dietary antioxidants [55,56]. Under the experimental conditions of this study, fermentation exerted a preservative effect on the inherent levels of vitamins C, A, and E, as well increased the bioavailability of the level of vitamin B_2_ and that of phenolics in *P. oleracea* juice. The protective effects of fermented *P. oleracea* juice against oxidative stress were investigated using Caco-2 cells line as a model to get information using an in vitro model closest to the in vivo conditions [11,13,14,15,16]. The intracellular accumulation of ROS was evaluated in order to assess the damage caused by induced oxidative stress and determine the capacity of the *P. oleracea* juice to counteract such damage. In our study, we found a significant decreased level of ROS in Caco-2 cells pre-treated with fermented *P. oleracea* juice compared to raw and chemically acidified *P. oleracea* juice.

On the other hand, inflammatory responses play a critical role in the pathogenesis of IBS. During the acute inflammatory reaction, clinical and experimental studies reported significant increases in the secretion of cyclooxygenases, inducible nitric oxide synthases (iNOS), and pro-inflammatory cytokines (e.g., interleukin-1β (IL-1β), interleukin-8 (IL-8), monocyte chemotactic protein-1 (MCP-1)), which promote inflammatory cell recruitment and activation [57,58,59,60,61]. The inhibition of the intestinal inflammation is one of the main research targets for IBS treatment [62]. Fermented *P. oleracea* juice strongly counteracted the inflammatory processes in Caco-2 cells treated with the inflammatory stimulus. The treatment with fermented juice limited the production of prostaglandin E2 by cyclooxygenase enzymes and nitric oxide by iNOS [63,64,65,66]. Synthesis of IL-8 and MCP-1 was also markedly counteracted by the treatment with the fermented *P. oleracea* juice. The role of MCP-1 and IL-8 in the pathophysiology of IBS has been widely recognized making them targets for anti-inflammatory treatments [67,68,69,70,71,72]. IL-8 and MCP-1 act as chemo-attractants that mediate neutrophil migration across the epithelial barrier [71,72,73,74,75]. The migration of a large number of neutrophils could damage junction proteins, thereby decreasing transepithelial resistance and increasing epithelial permeability [76,77]. Gut barrier dysfunction and the increase of intestinal paracellular permeability to noxious luminal substances in IBS is closely associated with the increase of pro-inflammatory cytokines [78,79,80]. Under the condition of this study, the fermented *P. oleracea* juice preserved the integrity of the Caco-2 cell monolayers. Although the study was carried out in vitro on Caco-2 cells, this type of cells is widely used as a model for the intestinal epithelial barrier because they form a monolayer and tight junction complexes characteristic of enterocytes [11,13,14,15,16]. Consequently, the consumption of fermented *P. oleracea* juice might contribute to maintaining the integrity of the intestinal barrier in vivo in IBS subjects. Filannino et al. [10] showed that lactic fermentation of *P. oleracea* puree dramatically increased the levels of linalool. This was shown to exert anti-inflammatory activity, thus might underlie the protective effects of fermented *P. oleracea* juice [81]. Furthermore, Filannino et al. [10] speculated that the bioconversion of oleraceins might be the reason for the increased antioxidant activity in fermented *P. oleracea* puree. In light of these findings, *P. oleracea* fermentation might be exploited to develop dietary supplements and pharmaceutical preparations, in addition to functional beverages. 

**Figure 6:**
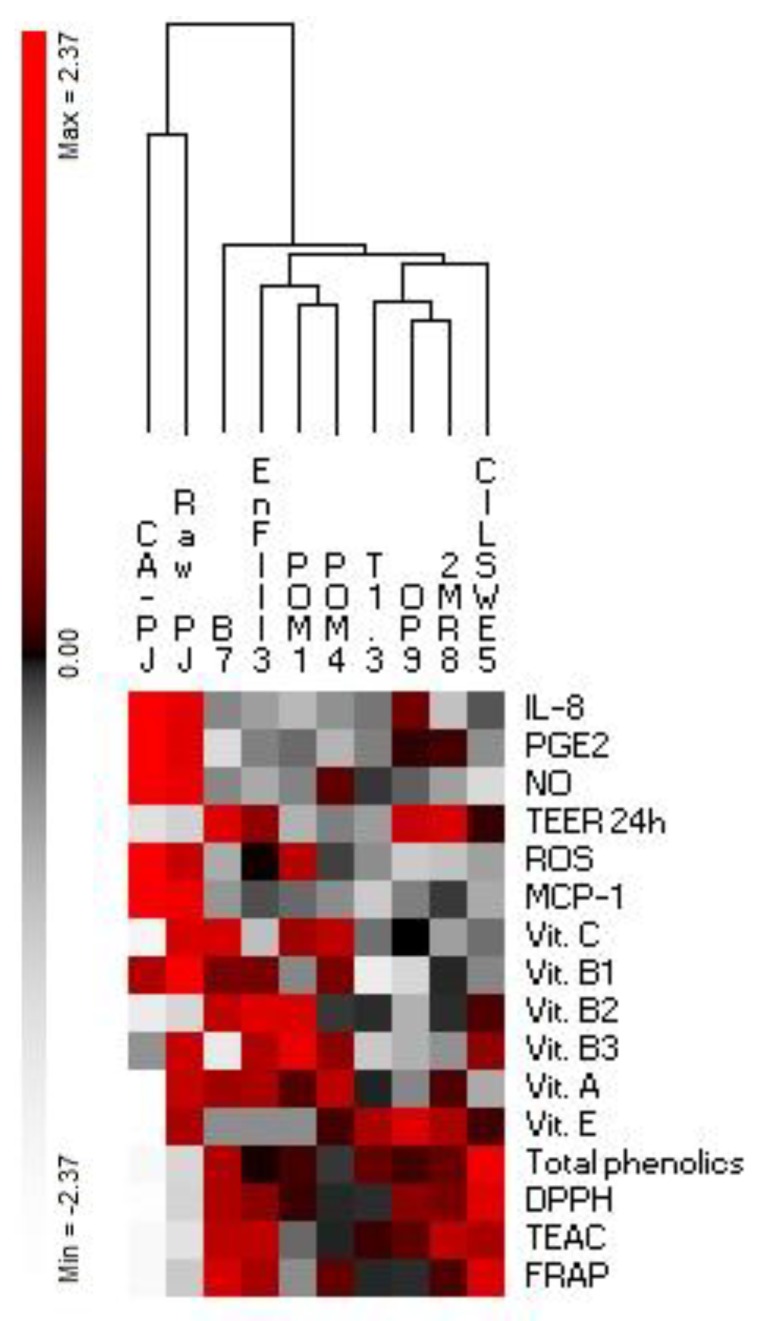
Permutation analysis of functional activities of raw *Portulaca oleracea* L. juice (Raw-PJ), chemically acidified PJ (CA-PJ), and PJs fermented (30 °C for 36 h) by *Lactobacillus plantarum* POM1, T1.3, and EnFIII3, *Lactobacillus brevis* POM4, *Lactobacillus rossiae* 2MR8, *Pediococcus pentosaceus* CILSWE5, *Leuconostoc mesenteroides* OP9, and *Lactobacillus kunkeei* B7. Normalized data for antioxidant activities (DPPH, TEAC, and FRAP) and levels of bioactive compounds (vitamins and total phenolics) in PJs, and pro-inflammatory mediators (PGE2, NO, IL-8, and MCP-1), reactive oxygen species (ROS), and transepithelial electrical resistance (TEER) levels in Caco-2 cells treated with PJs were subjected to permutation analysis.

Our study used a diversified spectrum of lactic acid bacteria species to investigate the effects of lactic fermentation on the restoring properties of *P. oleracea* L. juice. Some effects appeared to be strains- or species-specific, see Figure 6. For instance, the highest transepithelial electrical resistance values were observed with the juice fermented by *L. kunkeei* B7, *L. rossiae* 2MR8, and *Leuconostoc mesenteroides* OP9. In conclusion, considering the totality of the descriptors that we analyzed, we can summarize that the fermentation of *P. oleracea* L. juice with *L. kunkeei* B7 guaranteed the best combination for the content of bioactive compounds (free phenolics and vitamins), the ability to preserve the integrity of the intestinal barrier and to counteract the intestinal inflammation and the oxidative damage, see Figure 6. A validation of our findings regarding the functional features of fermented *P. oleracea* L. juice will be further performed through controlled in vivo experiments. Our work represents a pioneering study on the exploitation of *P. oleracea* L. juice as a functional beverage. Further studies might continue along this track and employ fermentation-based biotechnologies to improve other nutritional and functional features, as well as technological and sensorial properties of *P. oleracea* L. juice [22,82,83,84]. Since a probiotic potential has been proposed for most of the lactic acid bacteria species we used in this study [85,86,87,88,89,90,91,92,93,94,95,96,97,98,99,100], fermentation of *P. oleracea* L. juice might be also proposed to develop probiotic beverages. Within this perspective, synergistic effects between juice and probiotic bacteria might come to light [101].

## Figures and Tables

**Table 1:** Lactic acid bacteria strains (*n* = 8) used in this study.

Strain	Accession Number *	Reference
*Lactobacillus plantarum* POM1	MF967220	[17]
*L. plantarum* T1.3	MF967221	[18]
*L. plantarum* EnFIII3	n.a.	[19]
*Lactobacillus brevis* POM4	MF967222	[17]
*Lactobacillus rossiae* 2MR8	MF967223	[20]
*Pediococcus pentosaceus* CILSWE5	MF967224	[21]
*Leuconostoc mesenteroides* OP9	MF967225	[22]
*Lactobacillus kunkeei* B7	KX833124	[23]

* The 16S rRNA gene sequences were deposited in GenBank under the accession numbers shown. n.a., not available.

**Table 2:** Level of vitamins and total phenolics (mg per 100 mL of PJ) in raw *Portulaca oleracea* L. juice (Raw-PJ), chemically acidified PJ (CA-PJ), and PJs fermented at 30°C for 36 h by *Lactobacillus plantarum* POM1, T1.3, and EnFIII3, *Lactobacillus brevis* POM4, *Lactobacillus rossiae* 2MR8, *Pediococcus pentosaceus* CILSWE5, *Leuconostoc mesenteroides* OP9, and *Lactobacillus kunkeei* B7.

	mg per 100 mL of PJ						
	Ascorbic Acid (Vitamin C)	Thiamin (Vitamin B_1_)	Riboflavin (Vitamin B_2_)	Niacin (Vitamin B_3_)	β-Carotene (Vitamin A)	α-Tocopherol (Vitamin E)	Total Phenolics (Gallic Acid Equivalents)
Raw–PJ	22 ± 2 ^a^	0.047 ± 0.004 ^a^	0.11 ± 0.004 ^d^	0.48 ± 0.02 ^a^	2.00 ± 0.02 ^a^	2.5 ± 0.1 ^a^	85 ± 6 ^bc^
CA–PJ	12 ± 1 ^d^	0.044 ± 0.003 ^a^	0.10 ± 0.002 ^d^	0.42 ± 0.02 ^c^	1.50 ± 0.03 ^d^	2.0 ± 0.1 ^b^	70 ± 2 ^d^
*L. plantarum* POM1	20 ± 2 ^a^	0.041 ± 0.002 ^a^	0.18 ± 0.004 ^a^	0.50 ± 0.01 ^a^	1.88 ± 0.02 ^b^	2.3 ± 0.1 ^a^	112 ± 4 ^b^
*L. plantarum* T1.3	17 ± 2 ^b^	0.038 ± 0.002 ^a^	0.15 ± 0.006 ^b^	0.40 ± 0.03 ^c^	1.85 ± 0.04 ^b^	2.5 ± 0.3 ^a^	115 ± 6 ^b^
*L. plantarum* EnFIII3	15 ± 2 ^c^	0.043 ± 0.005 ^a^	0.19 ± 0.005 ^a^	0.47 ± 0.02 ^a^	1.96 ± 0.04 ^a^	2.3 ± 0.1 ^a^	110 ± 3 ^b^
*L. brevis* POM4	21 ± 3 ^a^	0.043 ± 0.005 ^a^	0.14 ± 0.003 ^b^	0.46 ± 0.02 ^b^	1.99 ± 0.03 ^a^	2.4 ± 0.2 ^a^	108 ± 4 ^b^
*L. rossiae* 2MR8	16 ± 3 ^c^	0.042 ± 0.004 ^a^	0.15 ± 0.004 ^b^	0.42 ± 0.02 ^c^	1.88 ± 0.03 ^b^	2.5 ± 0.1 ^a^	115 ± 8 ^b^
*P. pentosaceus* CILSWE5	17 ± 2 ^b^	0.041 ± 0.002 ^a^	0.15 ± 0.005 ^b^	0.46 ± 0.03 ^b^	1.75 ± 0.04 ^c^	2.4 ± 0.2 ^a^	145 ± 8 ^a^
*Leuc. mesenteroides* OP9	18 ± 2 ^b^	0.039 ± 0.002 ^a^	0.12 ± 0.004 ^c^	0.41 ± 0.02 ^b^	1.79 ± 0.02 ^c^	2.6 ± 0.3 ^a^	112 ± 7 ^b^
*L. kunkeei* B7	22 ± 2 ^a^	0.043 ± 0.002 ^a^	0.17 ± 0.002 ^a^	0.38 ± 0.01 ^c^	1.95 ± 0.02 ^a^	2.3 ± 0.1 ^a^	125 ± 6 ^b^

Data are the means ± standard deviation of three biological replicates analyzed in triplicate. Means within a column with different superscript letters differ significantly (*p* < 0.05).
